# Letter from “Medicus”

**Published:** 1860-04

**Authors:** 

**Affiliations:** Milwaukee


MILWAUKEE, March 13, 1860.

*To the Editors of the Chicago Medical Journal:*

GENTLEMEN: I notice in the last number of the *Medical Examiner*, printed in your city that E. B. Woolcott, of this city, attempts to defend himself against the imputation resting upon him, of malpractice in the case of Page, the fireman injured at the Watertown accident, by raising a question of "medical ethics."

According to his own showing, he left Page as he found him. If it was a fracture why did he put on the mechanical power, and why did he leave it without the usual dressing?

If it was a dislocation, why did he not reduce it?

In regard to the question of ethics it is doubtful what claim Dr. Wolcott may have on the regular profession to be recognized. If, as is generally understood, he at one time left its ranks, and announced himself a disciple of Turner, author of the notorious book called, "Fallacies of the Faculty, and the "Chrono-Thermal System of Medicine," which he will hardly dare to deny—and if he has never publicly renounced this *ism*, it is but just that he should be called upon to *purge himself* of this contempt before appearing to urge a charge of violation of the code of ethics. The certificate of character volunteered by the *Examiner* is not sufficient. Let the Doctor himself say where he stands just now.

Respectfully,

MEDICUS.

---

☞ Any of our subscribers having a copy of "Todd's Cyclopedia of Anatomy and Physiology," can find a purchaser, if desired, by addressing the editors of the *Journal*.

---

☞ We are obliged to omit, until the next number, the names of the graduating class of Rush Medical College for the session of 1859–60.

